# Association between Statin Use and Survival in Cancer Patients with Brain Metastasis: Retrospective Analysis from the Chinese Population

**DOI:** 10.3390/ph15121474

**Published:** 2022-11-26

**Authors:** Yu Min, Zheran Liu, Zhigong Wei, Ruidan Li, Jing Jin, Yu Zhang, Xingchen Peng

**Affiliations:** 1 Department of Biotherapy and National Clinical Research Center for Geriatrics, Cancer Center, West China Hospital, Sichuan University, Chengdu 610041, China; 2 Department of Neurosurgery, Affiliated Hospital of Chengdu University, Chengdu 610045, China; 3 Department of Neurosurgery, West China Hospital, Sichuan University, Chengdu 610041, China

**Keywords:** statin, cancer, brain metastasis, overall survival, risk factor

## Abstract

Brain metastasis predicts a worse clinical outcome in cancer patients. Emerging observational evidence suggests that statin use has a protective role in overall cancer prevention. Whether statin use could also be a supplementary treatment for advanced-stage cancers remains under researched and controversial. Data for cancer patients with brain metastasis were selected from the linked electronic medical care records of the West China Hospital between October 2010 and July 2019. Fisher’s exact chi-square test was used to compare the differences between cohorts. Multivariate Cox analysis was conducted to adjust the potential confounders in evaluating the role of statin use in the overall survival (OS) of cancer patients with brain metastasis. There were 4510 brain metastatic patients included in this retrospective study. The overall statin use rate in our patients was 5.28% (219 cases/4510 cases). Compared with the non-statin use cohort, patients who received statin therapy showed a decreased Karnofsky performance score (KPS, *p* < 0.001) and lower high-density lipoprotein (HDL, *p* = 0.020) but higher body mass index (BMI, *p* = 0.002) and triglyceride (TG, *p* < 0.001) at admission. There was no association between statin use and the OS of the cancer patients with brain metastasis (Hazard ratio (HR) = 0.90, 95% confidence interval (CI): 0.73–1.07, *p* = 0.213) during the univariate analysis. However, after adjusting for baseline patient characteristics, metabolism indicators, and cancer-specific factors, statin use was shown to have a significant protective role, aiding the survival of the cancer patients with brain metastasis (_adjust_HR = 0.82, 95%CI: 0.69–0.99, *p* = 0.034). Our results highlight that statin use shows significant survival benefits in cancer patients with brain metastasis. However, future research is needed to validate our findings.

## 1. Introduction

Cancers with brain metastasis, which frequently arise in patients with lung cancer, breast cancer, as well as melanoma, indicate a worse prognosis [1]. According to the reports based on the Surveillance Epidemiology and End Results (SEER) analysis in 2016, the incidence proportion of brain metastases ranges approximately from 0.07% to 15.83% among the whole cohort within specific cancer sites [2]. However, the true rate has not been well evaluated, and the existing rate could be underestimated owing to many factors, such as the utility of screening imaging of the brain and patients who died prior to the diagnosis of brain metastasis [2,3].

Due to anatomical differences and the unique brain microenvironment, the current treatments are largely palliative in nature in most patients [3]. The distinct and profound selective pressure on cancer cells shapes the metastatic process and limits therapeutic responses [3]. Over the past few years, several targeted treatments have been applied to subgroups of patients with brain metastasis harboring specific molecular alterations (including, but not limited to, immune checkpoint inhibitors and specific gene mutation), which presented a remarkably improved overall prognosis [4,5,6]. However, most patients do not fall into these categories. Consequently, discovering more general alternative treatment strategies to prolong the survival of cancer patients with brain metastasis could better help the clinical management of this population.

Statins, as the inhibitors of 3-hydroxy-3-methyl-glutaryl-CoA (HMG-CoA) reductase [7], are one of the most frequently prescribed drugs for the clinical control of hypercholesterolemia. Recently, numerous studies have revealed that statins, followed by aspirin and metformin, also have a potentially protective role in cancer prevention and prognosis due to their ability to inhibit proliferation, angiogenesis, and inflammation via multiple molecular signaling pathways for cancer prevention [8,9,10]. Using clinical evidence from pooled meta-analysis based on retrospective cohort studies, Chen et al. [11] determined that statin use is associated with improved overall survival (OS, hazard ratio (HR) = 0.79), cancer-specific survival (CSS, HR = 0.83), and recurrence-free survival (RFS, HR = 0.85). Conversely, results from Lee et al. [12] revealed that statins could increase the risk of cancer mortality (HR = 1.33). Therefore, the role of statin use in cancer prevention and treatment needs further confirmation. Furthermore, most of the related studies were conducted in European populations from developed countries, and only a few of the studies were conducted in Asian regions (mainly in Japan, Korea, and Taiwan). Thus, a study using participants derived from the Chinese population might help to fill this gap and provide more insightful perspectives. Moreover, regarding the emerging therapeutic effects of statin use on primary brain tumors [13], whether statin use could also improve survival chances from secondary brain tumors is still unknown, and thus worth investigating further.

In the current study, we aim to first evaluate the role of statin use in the prognosis for cancer patients with brain metastasis in a large cohort from the Chinese population, which could help to provide more evidence for the efficacy of repurposing the lipid-lowering drug in cancer prevention and treatment.

## 2. Results

### 2.1. Demographic Clinical Characteristics of the Study Population

**Figure 1:**
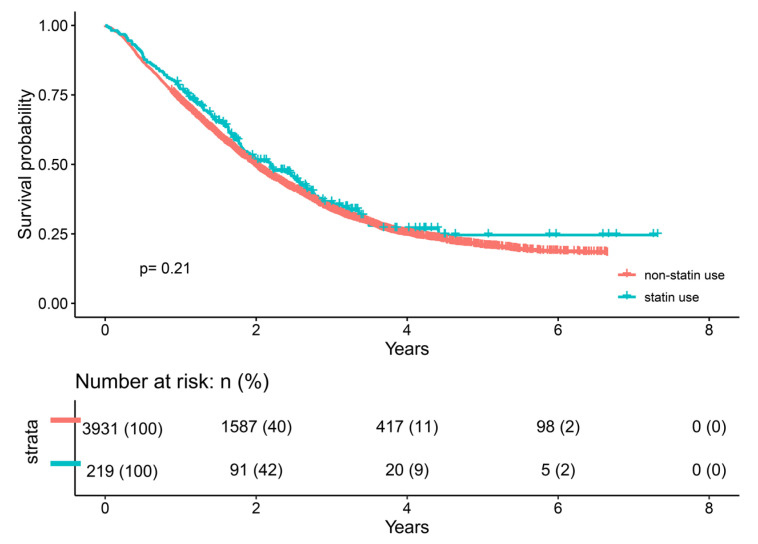
The Kaplan–Meier curve between statin use and overall survival of cancer patients with brain metastasis.

Generally, there were 4150 cancer patients with brain metastasis included in the present study. The mean age in the whole cohort was 56.83 years, 56.40 years in the non-statin cohort, and 64.53 years in the statin-use cohort (*p* < 0.001). There was no statistically significant difference in sex, the number of brain metastatic tumor sites, secondary malignancy record, LDL level, smoking and alcohol-intake status, and cancer-associated treatment indicators (all *p* > 0.05). The overall statin use rate in cancer patients with brain metastasis was 5.28% (219 cases/4150 cases). Notably, compared with the non-statin use group, patients who received statin therapy showed relatively lower KPS scores (*p* < 0.001), higher BMIs (*p* = 0.002), lower serum HDL levels (*p* = 0.020), and higher serum TG levels (*p* < 0.001). Patients with statin use showed a remarkably higher incidence of hypertension, diabetes, and hyperlipidemia conditions (all *p* < 0.001). The specific comparison between statin users and non-statin users is summarized in Table 1. The Kaplan–Meier curves showed that statins did not influence the long-term survival of brain metastatic patients (*p* = 0.21, Figure 1).

### 2.2. Univariate Cox Analysis

The univariate Cox analysis revealed that age at diagnosis (HR = 1.01, 95% confidence interval (CI): 1.00–1.01, *p* < 0.001), number of brain metastatic sites (HR = 1.03, 95% CI: 1.01–1.04, *p* < 0.001), secondary malignancy (HR = 1.14, 95%CI: 1.05–1.24, *p* = 0.002), smoking (ever: HR = 1.30, 95%CI: 1.19–1.42, *p* < 0.001; current: HR = 1.27, 95%CI: 1.13–1.42), and alcohol intake (HR = 1.21, 95%CI: 1.11–1.32, *p* < 0.001) were the potential risk factors in impairing the OS of brain metastatic patients. Conversely, female sex (HR = 0.81, 95% CI: 0.75–0.87, *p* < 0.001), KPS (HR = 0.98, 95%CI: 0.98–0.99, *p* < 0.001), BMI (HR = 0.97, 95%CI: 0.95–0.98, *p* < 0.001), HDL (HR = 0.76, 95%CI: 0.69–0.85, *p* < 0.001), LDL (HR = 0.93, 95%CI: 0.88–0.97, *p* = 0.002), TG (HR = 0.93, 95%CI: 0.89–0.98, *p* = 0.005), and cancer-associated treatment (radiotherapy: HR = 0.88, 95%CI: 0.82–0.95, *p* = 0.001; chemotherapy: HR = 0.84, 95%CI: 0.78–0.91, *p* < 0.001; targeted therapy: HR = 0.72, 95%CI: 0.66–0.78, *p* < 0.001) played a protective role in brain metastasis cancer patients. However, statin use was not statistically significantly associated with the prognosis of brain cancer patients (HR = 0.90, 95%CI: 0.73–1.07, *p* = 0.213) (Table 2).

### 2.3. Multivariate Cox Analysis

After adjusting the age, sex, serum indicators, comorbidities, cancer-associated factors, and treatments, statin use was markedly associated with an increased OS probability in brain metastatic patients (HR = 0.82, 95%CI: 0.69–0.99, *p* = 0.034). Meanwhile, higher KPS (HR = 0.99, 95%CI: 0.98–0.99, *p* < 0.001), BMI (HR = 0.97, 95%CI: 0.96–0.98, *p* < 0.001), serum levels of HDL (HR = 0.78, 0.70–0.87, *p* < 0.001), as well as TG (HR = 0.95, 95%CI: 0.90–1.00, *p* = 0.034) and the targeted therapy performed (HR = 0.76, 95%CI: 0.69–0.83, *p* < 0.001) were the independent protective factors in the OS of brain metastatic patients. On the other hand, elderly patients (HR = 1.01, 95%CI: 1.00–1.01, *p* = 0.014), multiple brain metastatic sites (HR = 1.02, 95%CI: 1.01–1.03, *p* < 0.001), the presence of secondary malignancy (HR = 1.23, 95%CI: 1.13–1.35, *p* < 0.001), and previously having smoked (HR = 1.25, 95%CI: 1.11–1.41, *p* < 0.001) predicted a worse chance of survival for cancer patients with brain metastasis.

To evaluate whether the non-statin variables also fit into the same model in the control population, the multivariate Cox analysis was reconducted, and the results were compared with the findings in the whole population (Supplementary Table S1). As expected, similar results were determined in the control population, which indicated the stability and rationale of the results. Additionally, the subgroup analysis showed there was no significant crossover effect of statins on interactions with other variables (Supplementary Figure S1).

## 3. Discussion

Brain metastasis is regarded as a severe condition in the progression of late-stage cancer [1,3]. Recent studies and comprehensive reviews have highlighted that novel treatment modalities are urgently needed for application to clinical practice to prolong the survival of this subpopulation. Drug repurposing has recently been regarded as a feasible way to overcome this challenge. Notably, compelling evidence has proven the protective role of statin use in the prevention of different site-specific cancers [14,15,16,17]. However, whether statin use could reduce cancer mortality in advanced-stage patients has rarely been explored, and the results are debatable. In particular, one nationwide population-based study (the Surveillance, Epidemiology and End Results, SEER) conducted by Lin et al. revealed that a significantly prolonged survival pattern was observed in elderly patients (>65 years) with stage IV NSCLC who received statin therapy [18]. Conversely, the results from Leigh et al.’s work did not support the preventive role of statin use in brain metastasis risk in lung cancer. Consequently, the role of statin use on late-stage lung cancer patients needs to be further explored.

In the present study, to the best of our knowledge, we are one of only a few studies evaluating the beneficial role of statin use in brain metastatic patients in the Asian population. The statin use rate in our study was 5.28% of the cancer patients with brain metastasis, which was significantly lower than the report from Lin et al.’s work (27%) focused on the stage IV NSCLC population [18]. The divergence could be attributed to the varied sample size and selected age group in the latter study. In our results, the statin use records were based on the electronic medical system, which could inevitably underestimate the real statin use rate in the population. In our findings, the univariate analysis showed that the current statin use was not associated with the prognosis of brain metastatic patients’ survival (HR = 0.90, 95%CI: 0.73–1.07, *p* = 0.213). By contrast, we found statin use could remarkably increase the survival of cancer patients with brain metastasis (_adjust_HR = 0.82, 95%CI: 0.69–0.99, *p* = 0.034), after adjusting other confounders. In our study, compared with the non-statin-use group, statin-use patients showed elderly age at diagnosis, lower KPS, abnormal lipid metabolism, and more concurrent comorbidities. Thus, the imbalance of the two groups could obfuscate the association between statin use and cancer survival during the univariate analysis.

Reviewing previous research focusing on statin use and cancer prevention and associated survival shows that the protective effects of statin use were also observed in cancer patients with varied specific sites. For instance, in one pan-cancer analysis study, Wang et al. demonstrated that current statin use is associated with a significantly decreased risk of cancer mortality (HR = 0.78, 95%CI: 0.71–0.86) in postmenopausal women, regardless of potency, lipophilicity, type, or duration [19]. Several more recent studies have confirmed the optimal effect of statin use in decreasing the metastasis risk and increasing the survival probabilities of melanoma patients [20,21,22]. Notably, Yu et al. [21] identified that HMG-CoA reductase inhibitors, referred to as statins, might prevent melanoma metastasis by using the computational drug repositioning system. Moreover, similar to the genetic association they determined, statin use could halve the risk of metastasis of melanoma (_adjust_odd ratio (OR) = 0.48). However, the available studies focusing on the effects of statin use on survival outcomes of metastatic melanoma patients were scarce. Only a few preclinical studies provided evidence for supporting statin use in metastatic melanoma [23,24]. Some potential signature pathways (Rho/Rho-associated coiled-coil-containing protein kinase pathways) participate in reducing distant metastasis, cell invasion, and adhesion in mouse models.

Clinically, our results filled this research gap and supported the beneficial role of statin use in late-stage cancer patients in the Chinese population. Nonetheless, in one prospective RCT for evaluating additional statin use on the survival of stage IV cancer patients receiving whole-brain radiotherapy (WBRT), there was no statistically significant beneficial role in improving the 1-year OS or progress-free survival (PFS) observed during the analysis [8]. Yet, some limitations in their study need to be pointed out. As they mentioned in the text, although the prospective RCT design strengthened the evidence of the study, only twenty-seven brain metastatic patients (13 patients in the control group and 14 patients in the simvastatin group) were ultimately involved in the follow-up evaluation. Interestingly, some recent studies highlighted that the different statin use adherence was also associated with the all-cause mortality of cancer patients. Based on a similar East Asian population, Lee et al. [12] discovered that poor adherence was associated with an increased risk of cancer mortality (HR = 1.33, 95% CI: 1.16–1.52). Thus, the detailed dose, adherence, and duration of the statin use of each patient could help to find more connections between statin use and advanced cancer prognosis in prospective research.

An increasing number of preclinical data indicates that statins may have powerful antitumor effects [7,13]. In line with the clinical observations on statin use and better cancer survival, some molecular changes might provide the underlying mechanisms and evidence of this phenomenon [9,10]. For example, Yao et al. determined that simvastatin, one type of statin, could kill triple-negative breast cancer (TNBC) cells in vivo and in vitro (MDA-MB-231) by inducing strong ferroptosis [10]. Furthermore, the cytotoxicity effects of statin use were also observed in metastatic lung cancer cells. Notably, results from the team of Luttman et al. revealed that a combination of simvastatin and ABL kinase allosteric inhibitors could enhance the apoptosis in metastatic lung cancer cells (PC9 BrM3) via the mevalonate (MVA) pathway. Meanwhile, in brain metastatic mouse models, they further confirmed that combination therapy impaired the metastatic colonization with subsequently increased survival [9]. In addition, some earlier studies have also confirmed that these anti-metastatic effects could also contribute to the reduction in E-selectin, an endothelial leukocyte adhesion molecule, and the attenuation of TNF-α in tumor cell invasion [25]. In one of the latest comprehensive reviews for summarizing the molecular pathways of statin-mediated anticancer effects in lung cancer [26], researchers highlighted the varied pathways of different statins used in lung cancer. On the one hand, simvastatin was determined to enhance apoptosis in cancer cells and the degradation of p53 mutant, which helps to inhibit the distant metastasis of lung cancer [27]. Additionally, simvastatin could also activate the AMPK/Akt/mTOR signaling pathway and, correspondingly, reduce the oncogenic transformation through metabolic modulation. On the other hand, the bioactivity of lovastatin was recently found to reduce the expression of matrix metalloproteinase (MMP)-9 and MMP-2, as well as the suppression of the Ras isoprenylation, which could subsequently decrease the invasive ability of tumor cells [28,29]. Thus, combined with the findings which were observed in our study, statin therapy might be a promising way to increase survival for cancer patients with brain metastasis, but future work is needed to better clarify the underlying mechanisms in distant antitumor effects.

Notably, we constructed a large-scale Chinese brain metastatic cohort for evaluating the prognostic effects of statin use on cancer patients with brain metastasis, which was the first strength of our study. Additionally, we controlled for a number of clinicopathological confounders to reduce the bias in verifying the role of statin use in cancer survival. Meanwhile, we also confirmed several independent predictive prognostic factors in brain metastasis patients, including age at diagnosis, number of metastatic sites, smoking status, KPS, secondary malignancy, and lipid metabolism indicators and treatment strategies. In particular, receiving targeted therapy was the strongest predictor, which could remarkably improve the survival probability of brain metastasis patients by nearly 24%. Compelling evidence has demonstrated that being overweight is a pivotal risk factor for the occurrence of common cancers [30,31]. Interestingly, higher BMI and TG levels showed a significant correlation with the survival of brain metastatic patients in the current study. Although maintaining a higher BMI suggests an adequate nutrient condition, since weight loss or cachexia are frequently presented in cancer patients, it is hard to draw conclusions about the association between BMI and TG levels and the survival of patients who have been given statins.

Nevertheless, there are some limitations that need to be mentioned, which are expected to be addressed in later work. First, the study design was a retrospective hospital-based model, which could inevitably lead to selection bias, regardless of the promising sample size involved. Second, although we confirmed the protective role of statin use in the prognosis of general brain metastatic patients, whether statin use could also improve survival in each primary cancer needs to be further investigated. Finally, the proportion of brain metastatic patients receiving statin therapy was relatively small, and atorvastatin is the most frequently prescribed type of the drug in our hospital. Thus, the statins used were not divided into different subtypes for further analysis. Therefore, future preclinical experiments studies focused on underlying mechanisms of statin use on secondary brain malignancies microenvironment and clinical trials with prospective randomized controlled designs could provide more robust evidence for guiding statin repurposing in cancer prevention and treatment.

## 4. Materials and Methods

### 4.1. Data Source

The clinicopathological characteristics and follow-up data of the cancer patients were retrospectively reviewed and extracted from the linked electronic medical care records in the West China Hospital between October 2010 and July 2019. The medical records of the West China Hospital are a large-scale, population-based database, which contains the baseline information, clinicopathological features, treatment modalities, and follow-up information for patients [32,33]. The study was conducted according to the guidelines of the Declaration of Helsinki. The ethical approval was given by the West China Hospital Ethics Committee. The reporting of the present study followed the STROBE statements [34].

### 4.2. Patient Selection

**Figure 2:**
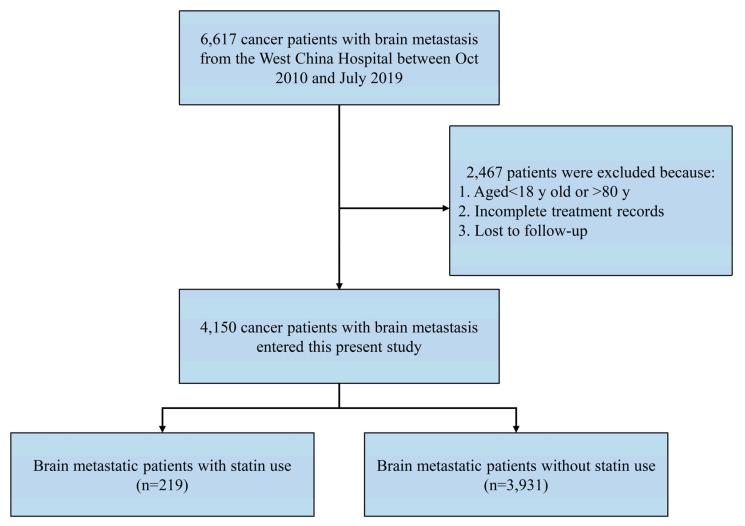
The flow diagram in the patient selection process.

We included cancer patients with brain metastasis in the present study. The diagnosis of brain metastasis was based on brain imaging findings [35,36]. The exclusion criteria were as follows: (1) patients aged <18 years or >80 years; (2) incomplete cancer-associated treatment information; and (3) unknown survival information. The detailed patient selection process is presented in Figure 2.

### 4.3. Clinical Variables Selection

Demographic clinical factors which were potentially associated with the survival outcomes [37,38,39] were collected, including age at diagnosis, sex (male and female), body mass index (BMI), Karnofsky performance score (KPS, a scale for evaluating the condition of cancer patients (ranging from 0 to 100); the higher the KPS score the patients obtained, the better health condition they were in), hypertension, diabetes, smoking (classified as never having smoked, having smoked in the past, and current smoker), alcohol, lipid metabolism indicators (high-density lipoprotein (HDL), low-density lipoprotein (LDH), triglyceride (TG), and hyperlipidemia). Statin use was ascertained from the electronic medication record system in our hospital. The proportion of statin therapy in the brain metastatic patients was relatively small and atorvastatin was the most frequently prescribed type in our hospital. Consequently, the statins were not divided into different subtypes for further analysis. The patients were classified as statin users if there was a record of statin use during their hospitalization and in their recent medical history.

### 4.4. Cancer-Related Factors

Based on the existing clinicopathological characteristics in our hospital, we collected the following cancer-related factors: the number of brain metastatic sites and the records of secondary malignancy during follow-up. The treatment records were reviewed and mainly classified into five parts: craniotomy for brain metastatic sites, radiotherapy, chemotherapy, and targeted therapy records of brain metastatic sites.

### 4.5. Study Outcome

The primary outcome of our study was the OS in cancer patients with brain metastasis with statin use or not. The secondary outcome was to evaluate other potential prognostic factors in the OS of cancer patients with brain metastasis. Survival times were defined as the period from the date of diagnosis to the date of all-cause death, and alive patients were defined according to the death certifications we censored in April 2021.

### 4.6. Statistical Analysis

The sample size calculation was conducted using the software “PASS” (version 21.0.3, Utah, USA) [40,41]. Specifically, a two-sided log rank test with an overall sample size of 4138 subjects (3931 in the control group and 207 in the treatment group) achieves 95% power at a 0.05 significance level to detect a hazard ratio of 0.82 when the control group hazard rate is 1. Baseline characteristics according to statin use were compared by using the Pearson-chi square test or Fisher’s exact chi-square test (dependent on the expected value and the number of the cohorts), and quantitative variables (One-way ANOVA test). Univariate Cox analysis was conducted to evaluate the association between each factor involved and OS. Multivariate Cox analyses were used to evaluate the hazard ratio (HR) of statin use on the prognosis for cancer patients with brain metastasis after adjusting for other confounders. A two-tailed *p*-value of <0.05 was considered to be statistically significant. The analyses and the Kaplan–Meier curves were all conducted using the R 4.1.2 software (https://cran.r-project.org/), which were accessed on 19 September 2022.

## 5. Conclusions

In summary, we discovered the protective role of statin use in cancer patients with brain metastasis in the Chinese population, which could decrease the risk of all-cause mortality by approximately 18%. Future well-designed studies with larger sample sizes are warranted to validate our findings and provide more robust evidence for statin use in cancer treatment.

## Figures and Tables

**Table 1:** The clinical demographic characteristics of the brain metastatic cancer patients in the present study.

Variables	Subgroup	Overall (*n* = 4150)	Non-Statin Use (*n* = 3931)	Statin Use (*n* = 219)	*p*
Age ^a^	/	56.83 (11.77)	56.40 (11.67)	64.53 (10.87)	**<0.001** ^b^
Sex	Female	1817 (43.8)	1726 (43.9)	91 (41.6)	0.539 ^c^
KPS ^a^	/	79.04 (10.57)	79.18 (10.49)	76.48 (11.52)	**<0.001** ^b^
No.MT ^a^	/	4.99 (3.89)	5.00 (3.89)	4.87 (3.94)	0.664 ^b^
Secondary Tumor	Yes	2865 (69.0)	2711 (69.0)	154 (70.3)	0.729 ^c^
BMI ^a^	/	22.34 (3.19)	22.30 (3.18)	23.09 (3.29)	**0.002** ^b^
HDL ^a^	/	1.25 (0.39)	1.25 (0.39)	1.19 (0.39)	**0.020** ^b^
LDL ^a^	/	2.59 (0.81)	2.58 (0.80)	2.65 (1.02)	0.232 ^b^
TG ^a^	/	1.45 (0.89)	1.43 (0.86)	1.73 (1.30)	**<0.001** ^b^
Smoking	Never	2764 (66.6)	2632 (67.0)	132 (60.3)	0.228 ^c^
	Ever	928 (22.4)	870 (22.1)	58 (26.5)	
	Current	456 (11.0)	427 (10.9)	29 (13.2)	
	NA	2 (0.0)	2 (0.1)	0 (0.0)	
Alcohol	Yes	876 (21.1)	825 (21.0)	51 (23.3)	0.467 ^c^
Hypertension	Yes	659 (15.9)	554 (14.1)	105 (47.9)	**<0.001** ^c^
Diabetes	Yes	345 (8.3)	293 (7.5)	52 (23.7)	**<0.001** ^c^
Hyperlipidemia	Yes	101(2.4)	55 (1.3)	46 (21.0)	**<0.001** ^c^
Craniotomy	Performed	470 (11.3)	454 (11.5)	16 (7.3)	0.157 ^c^
Radiotherapy	Performed	1812 (43.6)	1706 (43.3)	106 (48.4)	0.146 ^c^
Chemotherapy	Performed	2587 (62.3)	2454 (62.4)	133 (60.7)	0.614 ^c^
Targeted Therapy	Performed	1239 (29.8)	1175 (29.8)	64 (29.2)	0.834 ^c^

Abbreviation: KPS: Karnofsky performance score; No.MT: number of brain metastatic sites; BMI: body mass index; HDL: High-density lipoprotein; LDL: low-density lipoprotein; TG: triglyceride; NA: not mentioned. ^a^ mean (SD) ^b^ One-way ANOVA test ^c^ Two-tail Fisher exact test. Bold values indicate statistical significance (*p* < 0.05).

**Table 2:** The univariate and multivariate Cox analyses of the statin use and overall survival of brain metastatic patients.

Variables	Subgroup	HR (95%CI)	*p*	HR (95%CI)	*p*
Statin use	No	Reference	0.213	Reference	**0.034**
	Yes	0.90 (0.73–1.07)		0.82 (0.69–0.99)	
Age	/	1.01 (1.00–1.01)	**<0.001**	1.01 (1.00–1.01)	**0.014**
Sex	male	Reference	**<0.001**	Reference	0.951
	female	0.81 (0.75–0.87)		1.00 (0.90–1.11)	
KPS	/	0.98 (0.98–0.99)	**<0.001**	0.99 (0.98–0.99)	**<0.001**
No. MT	/	1.03 (1.01–1.04)	**<0.001**	1.02 (1.01–1.03)	**<0.001**
Secondary malignancy	No	Reference	**0.002**	Reference	**<0.001**
	Yes	1.14 (1.05–1.24)		1.23 (1.13–1.35)	
BMI	/	0.97 (0.95–0.98)	**<0.001**	0.97 (0.96–0.98)	**<0.001**
HDL	/	0.76 (0.69–0.85)	**<0.001**	0.78 (0.70–0.87)	**<0.001**
LDL	/	0.93 (0.88–0.97)	**0.002**	0.97 (0.92–1.02)	0.181
TG	/	0.93 (0.89–0.98)	**0.005**	0.95 (0.90–1.00)	**0.034**
Smoking	never	Reference		Reference	
	ever	1.30 (1.19–1.42)	**<0.001**	1.25 (1.11–1.41)	**<0.001**
	current	1.27 (1.13–1.42)	**<0.001**	1.15 (0.99–1.32)	0.061
Alcohol	No	Reference	**<0.001**	Reference	0.489
	Yes	1.21 (1.11–1.32)		1.04 (0.93–1.16)	
Hypertension	No	Reference	1.000		
	Yes	1.00 (0.90–1.11)			
Diabetes	No	Reference	0.899		
	Yes	1.01 (0.88–1.16)			
Hyperlipidemia	No	Reference	0.151		
	Yes	0.83 (0.64–1.07)			
Craniotomy	No	Reference	0.761		
	Performed	0.94 (0.62–1.43)			
Radiotherapy	No	Reference	**0.001**	Reference	0.192
	Performed	0.88 (0.82–0.95)		0.94 (0.86–1.03)	
Chemotherapy	No	Reference	**<0.001**	Reference	0.071
	Performed	0.84 (0.78–0.91)		0.92 (0.83–1.01)	
Targeted Therapy	No	Reference	**<0.001**	Reference	**<0.001**
	Performed	0.72 (0.66–0.78)		0.76 (0.69–0.83)	

Abbreviation: HR: hazard ratio; CI: confidence interval; KPS: Karnofsky performance score; No.MT: number of brain metastatic sites; BMI: body mass index; HDL: High-density lipoprotein; LDL: low-density lipoprotein; TG: triglyceride. Bold values indicate statistical significance (*p* < 0.05).

## Data Availability

The datasets generated during and/or analyzed during the current study are available from the corresponding author upon reasonable request.

## Supplementary Materials

The following supporting information can be downloaded at: https://www.mdpi.com/article/10.3390/ph15121474/s1. Supplementary files: Figure S1. The subgroup analysis for evaluating the interactions between statin use and other covariates. Table S1. The multivariate Cox analysis of the association between covariates and survival of cancer patients with brain metastasis excluding the statin use population.
