# Supplementary material for: Association between Statin Use and Survival in Cancer Patients with Brain Metastasis: Retrospective Analysis from the Chinese Population
Source: Pharmaceuticals (Basel). 2022 Nov 26;15(12):1474. doi: 10.3390/ph15121474 (PMC9781124; doi:10.3390/ph15121474)
Supplement: Supplementary file 1 [file pharmaceuticals-15-01474-s001.zip › supplementary Table S1.pdf]

Table S1. The multivariate Cox analyses of the covariates and overall survival of brain metastatic patients in control population.

Variables	Subgroup	HR (95%CI)	***P***
Age	/	1.01(1.00-1.01)	**0.011**
Sex	male	Reference	0.563
	female	1.03(0.93-1.15)	
KPS	/	0.99(0.98-0.99)	**<0.001**
No. MT	/	1.02(1.01-1.03)	**0.001**
Secondary malignancy	No	Reference	
	Yes	1.22(1.11-1.33)	**<0.001**
BMI	/	0.97(0.95-0.98)	**<0.001**
HDL	/	0.76(0.68-0.86)	**<0.001**
LDL	/	0.96(0.912-1.02)	0.162
TG	/	0.95(0.90-1.00)	0.052
Smoking	never	Reference	
	ever	1.16(1.00-1.34)	**0.001**
	current	1.28(1.13-1.45)	
Alcohol	No	Reference	
	Yes	1.05(0.94-1.18)	0.372
Radiotherapy	No	Reference	0.104
	Performed	0.93(0.85-1.01)	
Chemotherapy	No	Reference	
	Performed	0.93(0.84-1.03)	0.152
Targeted Therapy	No	Reference	**<0.001**
	Performed	0.76(0.69-0.83)	

Abbreviation: HR: hazard ratio; CI: confidence interval; KPS: Karnofsky performance score; No.MT: number of brain metastatic sites; BMI: body mass index; HDL: High-density lipoprotein; LDL: low-density lipoprotein; TG: triglyceride.

Bold values indicate statistical significance (p<0.05).
