# Supplementary figures and images for: Association between Statin Use and Survival in Cancer Patients with Brain Metastasis: Retrospective Analysis from the Chinese Population
Source: Pharmaceuticals (Basel). 2022 Nov 26;15(12):1474. doi: 10.3390/ph15121474 (PMC9781124; doi:10.3390/ph15121474)

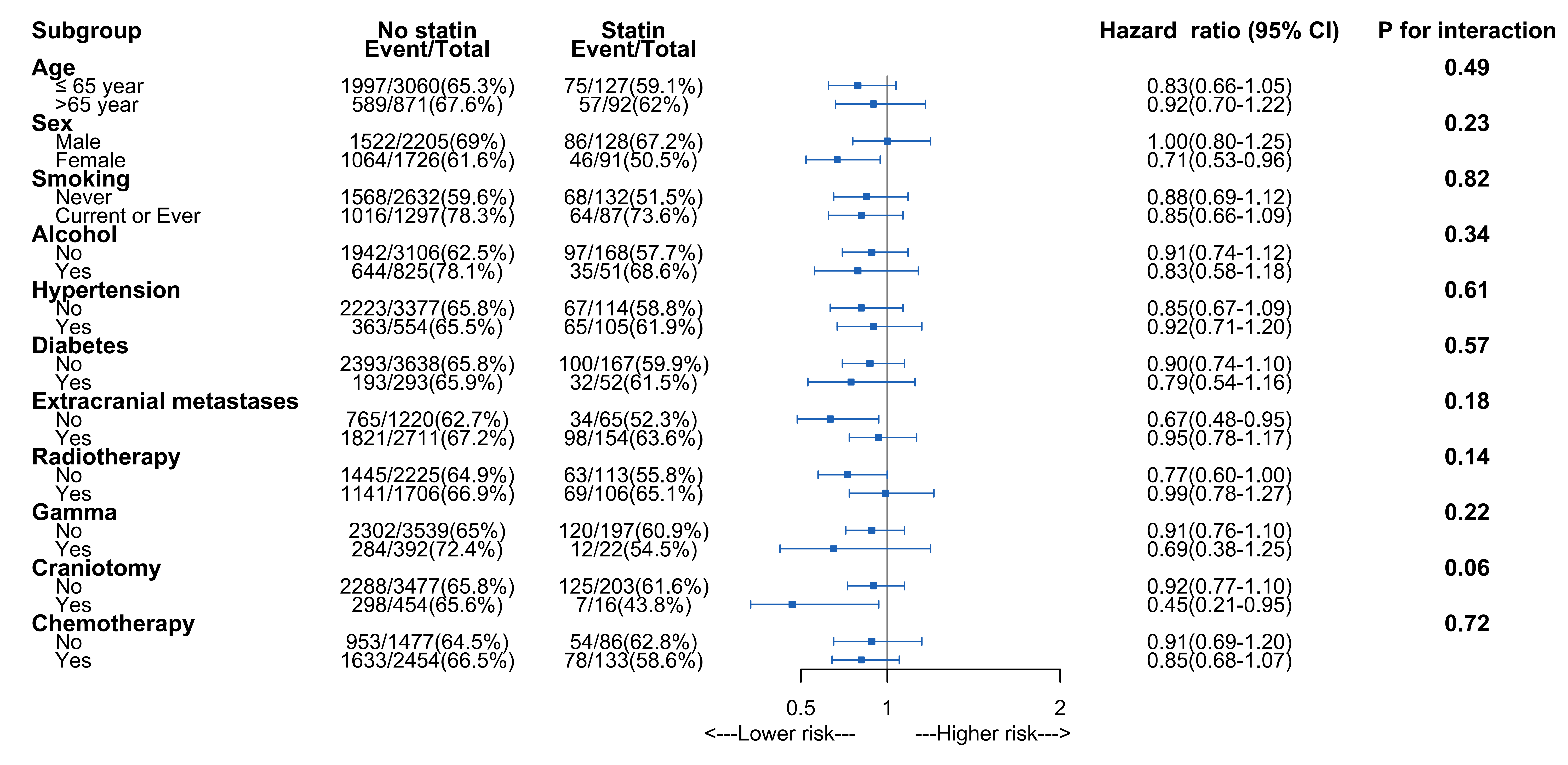

Supplement: Supplementary file 1 [file pharmaceuticals-15-01474-s001.zip › Figure S1.tif]
